# An Experimental and Numerical Study of Repairs on Composite Substrates with Composite and Aluminum Doublers Using Riveted, Bonded, and Hybrid Joints

**DOI:** 10.3390/ma12182978

**Published:** 2019-09-14

**Authors:** Siddharth Pitta, Francesc Roure, Daniel Crespo, Jose I. Rojas

**Affiliations:** 1 Department of Physics—Division of Aerospace Engineering, Universitat Politècnica de Catalunya, c/Esteve Terradas 7, 08860 Castelldefels, Spain; 2 Department of Strength of Materials and Structural Engineering, Universitat Politècnica de Catalunya, av. Diagonal 647, 08028 Barcelona, Spain; 3 Department of Physics, Barcelona Research Centre in Multiscale Science and Technology and Institut de Tècniques Energètiques, av. Eduard Maristany 16, 08019 Barcelona, Spain

**Keywords:** carbon fiber reinforced epoxy composite, substrate, rivets, adhesive bond, hybrid, aluminum alloy, strength

## Abstract

In this work, experimental and numerical analyses of repairs on carbon fiber reinforced epoxy (CFRE) substrates, with CFRE and aluminum alloy doublers typical of aircraft structures, are presented. The substrates have a bridge gap of 12.7 mm (simulated crack), repaired with twin doublers joined with riveted, adhesive bonded, and hybrid joints. The performance of the repairs using different doubler materials and joining techniques are compared under static loading. The experimental results show that riveted joints have the lowest strength, while adhesive bonded joints have the highest strength, irrespective of the doubler material. Finite element analysis (FEA) of the studied joints is also performed using commercial FEA tool Abaqus. In the FEA model, point-based fasteners are used for the rivets, and a cohesive zone contact model is used to simulate the adhesive bond. The FEA results indicate that the riveted joints have higher tensile stresses on the metal doublers compared to the composite doublers. As per the failure modes, interestingly, for hybrid joints using composite doublers, the doublers fail due to net-section failure, while, for hybrid joints using metal doublers, it is the composite substrate that fails due to net-section failure. This suggests vulnerability of the composite structures to mechanical fastener holes. Lastly, the Autodesk Helius composite tool is used for prediction of first-ply failure and ply load distribution, and for progressive failure analysis of the composite substrate.

## 1. Introduction

There is intense competition among aircraft manufacturers, from a structural point of view, to maximize the operational efficiency by utilizing materials with high strength-to-weight ratios. Metals, being traditionally the most used materials in aircraft, have been replaced progressively by composite materials. The last generation aircraft such as Airbus A350XWB and Boeing 787 have nearly 50% of the structure made of composite materials [1]. Their high strength-to-weight ratio and low density has caught the attention of manufacturers, driving the shift from metals to composites. If parts made of composite and/or metal are not co-cured or fabricated in a single-piece, then these multi-component structures need to have joints. Likewise, joints are essential in repairs and reinforcements. Hence, the structural integrity of all such structures depend on the strength of the joints [2]. Thus, understanding the joint behavior under different loading conditions is essential to certify joints, so that they can be used in aircraft structural maintenance.

There are many types of joining techniques and their applications are based on the required strength of the joints. The most common techniques are mechanical fasteners (e.g., rivets, bolts, pins, and screws), adhesive bonding, and hybrid joining [3,4,5]. On one side, the great advantage of mechanical fasteners is their ease of assembling and disassembling while allowing for reliable inspection. Despite these advantages, composite structures are more vulnerable to the high stress concentrations around fastener holes compared to ductile metals [6]. Aniello et al. [7] presented experimental investigations on shear behavior of riveted connections in steel structures, considering various parameters such as load eccentricity, variation in net-area, plate width, and number of rivets. Their results showed a considerable out-of-plane deformation for unsymmetrical joints, where the effects of bending were confined to the regions where plate discontinuities occurred. Jiang et al. [8] studied the corrosion characteristics of CFRP/Al riveted structures and analyzed the relationship between the microstructure and mechanical properties of these joints. Their results showed that the corrosion pits on Al sheet were mainly observed at the edges of the overlap area, which was due to larger potential difference between CFRP and Al. Li et al. [9] investigated the static and dynamic behavior of composite riveted joints under tension and observed that the total energy absorption of the composite riveted joint increases with an increasing loading rate. A model of the process of cold-driven rivets was investigated by Deng et al. [10], mainly to identify the origin and magnitude of the resulting clamping force exerted by the rivet on a lapped sheet.

On the other hand, adhesive bonded joints have gained more attention because of their high strength, uniform load distribution, and cost-effectiveness [11,12,13]. In practical applications, adhesive bonded joints have high stress concentrations at the ends of the overlap area, where maximum shear stresses occur compared to middle regions of the overlap. Peeling stresses are also higher at the ends of the overlap, and may cause the adhesive bonded joint to fail [14]. The thickness and type of adhesive play a major role in achieving high joint strengths, which have been studied by various researchers [15,16,17]. The quality of the adherend manufacturing process and the overlap length of the adhesive bond are also important in the design of these joints [18]. The stress concentration at the ends of the overlap can be reduced by increasing the overlap length up to a certain magnitude, beyond which, it no longer has an effect [18]. Hao et al. [19] investigated the behavior of CFRP-steel bonded joints at elevated temperatures, where significant effects of temperature-dependent bond-slip relation were observed under mode II loading. Li et al. [11] studied pre-preg bonded composite single-lap joints. The failure modes of adhesive bonded joints depend on the type of adhesive (brittle or ductile). Meng et al. [20] studied adhesively bonded steel butt joints under tension, torsion, and combined loading and reported failure modes and envelopes. Their experimental results showed that the failure mode of adhesive bonded steel joints shifts from a cohesive failure mode with subsequent adhesion failure under pure-torsion loading to a tensile cohesive failure under pure tension. Olcay et al. [21] validated a simple but effective design to improve the strength of thick adhesive composite strap joints with experiments and finite element analysis (FEA). Ribeiro et al. [22] studied the damage analysis of composite-aluminum adhesive bonded single-lap joints and discussed the FEA of adhesive bonded joints with cohesive zone models (CZM). Similarly, Ye et al. [23] studied the tensile failure behavior of adhesive-bonded composite single-lap joints with three-dimensional explicit FEA. Moura et al. [24] studied cohesive and continuum damage models applied to fracture characterization of bonded joints under loading in pure mode I and mode II.

Lastly, hybrid joint techniques involve the use of both adhesive bonding and mechanical fasteners [2,6,25,26,27]. Hybrid joints have different failure modes and most of them are due to debonding. Non-linear analysis of bonded and bolted joints by Hart-Smith [6] suggested that there is no significant advantage of hybrid joining over adhesive bonding in well-designed structures, but it may prevent defects and damage propagation. Our previous investigation on riveted, adhesive bonded, and hybrid repairs on metal substrates with metal and carbon fiber reinforced epoxy (CFRE) doublers showed that adhesive bonded and hybrid joints have nearly three times higher strength than riveted joints [28].

Hence, the motivation of this research is to study the performance of repairs on CFRE substrates, based on their peak strength under static loading, for the different types of joining techniques that can be implemented in airframe repairs. For this purpose, we present experimental and numerical analyses of discontinuous CFRE substrates repaired with twin doublers (case 1: with composite doublers, and case 2: with aluminum alloy doublers), joined with riveted, adhesive bonded, and hybrid joints, under tensile loading. In addition, numerical results on the behavior of these joints are obtained with commercial FEA code Abaqus computational aided engineering (CAE). Moreover, Helius composite tool from Autodesk (USA) is used for prediction of first-ply failure, ply load distribution, progressive failure, and failure envelopes of the composite substrate.

## 2. Materials and Methods

### 2.1. Properties of Materials

Composite substrates and doublers were made from plain 3K 200gsm carbon fiber fabric with SR 8100 epoxy system, manufactured and supplied by Composites Ate, S.L. (Barcelona, Spain). SR 8100 is a two-component epoxy system specially formulated for resin transfer processes such as injection or infusion. The cured system gives temperature resistance up to 80 °C (353 K). The composite substrate and doubler plates were machine cut to dimensions of 226 mm × 25.4 mm × 3 mm for substrates, 215.9 mm × 25.4 mm × 1.5 mm for doubler 1, and 165.1 mm × 25.4 mm × 1.5 mm for doubler 2. Substrates were made of 12 layers with stacking sequence [(45/−45), (0/90), (0/90), (0/90), (0/90), (45/−45)]s, while doublers were made of six layers with sequence [(45/−45), (0/90), (0,90)]s. The properties of the CFRE lamina are shown in Table 1. Glass fiber tabs were adhesively bonded to the ends of the substrates for smooth load transfer and for preventing failure at the grips/clamps. The tabs were made of epoxy resin reinforced with 0/90 glass fiber fabric, with dimensions 80 mm × 25.4 mm × 2.5 mm.

The metal doublers were made from a sheet of commercial aluminum alloy (AA) 2024-T3. The T3 temper consists in solution heat-treatment at 480 °C (753 K) for 1 h, followed by rapid water quenching to room temperature, cold working, and natural aging. The doublers were machine cut and supplied by Kaiser Aluminum (Spokane, WA, USA). The lay-out dimensions of the metal doublers are identical to those of the composite doublers: 215.9 mm × 25.4 mm × 1.59 mm for doubler 1, and 165.1 mm × 25.4 mm × 1.59 mm for doubler 2 (note that these doublers are a bit thicker). Table 2 and Table 3 show the properties and composition of this alloy, respectively, as provided by the manufacturer.

Adhesive bonded joints were prepared using Araldite 2031, which is a two-part thixotropic adhesive that consists of resin and hardener. Araldite 2031 is a commercial toughened adhesive with high chemical resistance and low shrinkage. The best performance can be achieved when the bond is cured for 16 h at 40 °C (313 K). The mechanical properties of Araldite 2031 are shown in Table 4, as provided by the manufacturer. On the other hand, the head of the blind rivets used for the fabrication of the riveted and hybrid joints is made of aluminum 1050A with a steel pin manufactured by Rapid Rivets (Hestra, Sweden). Typical aluminum 1050A rivets have an elastic modulus of 70 GPa with Poisson’s ratio of 0.3.

### 2.2. Specimen Preparation and Test Conditions

**Figure 1:**
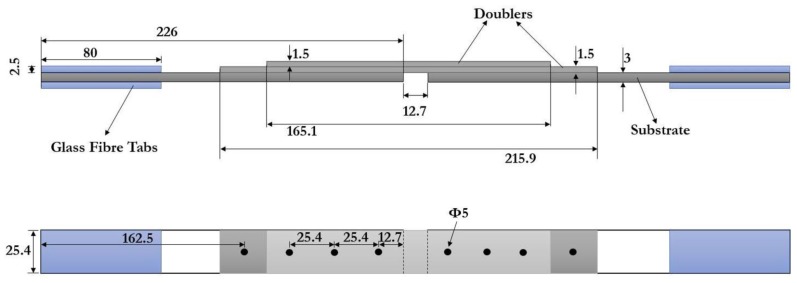
Dimensions of the studied double-lap joints (all units are in mm).

**Figure 2:**
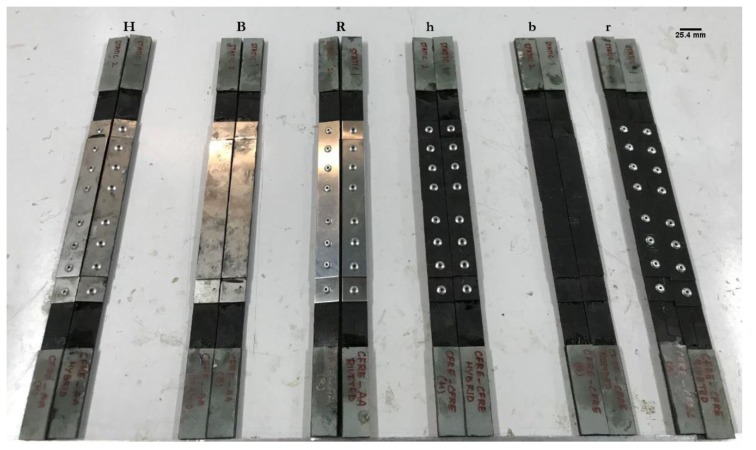
From left to right, hybrid (**H**), adhesive bonded (**B**), and riveted (**R**) joints of composite substrate with metal doublers, and hybrid (**h**), adhesive bonded (**b**), and riveted (**r**) joints of composite substrate with composite doublers.

Specimens of riveted, adhesive bonded, and hybrid joints of composite-composite (CFRE-CFRE) and composite-metal (CFRE-AA 2024 T3) were evaluated experimentally in monotonic tension tests. Since this is a comparative study between composite and metal doublers under various joining techniques, three specimens of each of the six joint types were tested. Riveted joints were fabricated by making holes of a 5-mm diameter at specific locations in the substrate and doublers (see Figure 1 and Figure 2). Holes were made with a carbide drill bit to have a perfect hole without irregularities around the fastener hole. A pop rivet gun was used to fasten rivets. Riveted and hybrid specimens have eight rivets fastened in a single row, and riveted joints were designed in accordance with Federal Aviation Administration (FAA) regulations [29]. These FAA standards establish a minimum pitch of three rivet diameters (recommended from four to six rivet diameters) between rivets, and a rivet-edge distance of 2.5 rivet diameters. Particularly, the specimens in this study have a pitch of 5.3 rivet diameters and rivet-edge distance of 2.5 rivet diameters. These same specifications were considered for preparing the hybrid joint specimens.

A thin layer of Araldite 2031 was applied between the surfaces of substrate and doublers for preparation of specimens with adhesive bonded and hybrid joints. The contact surfaces were abraded previously with sandpaper with a grit size of 80, and cleaned with acetone to remove any traces of dirt. These specimens were cured at 40 °C (313 K) for 16 h to obtain the best possible adhesive strength. Hybrid specimens were prepared by simply putting fasteners to bonded specimens after curing.

Lastly, the three specimens of each of the six studied joint types, as shown in Figure 2, were tested under monotonic tensile loading using a Metrotest 810 universal testing machine (UTM), controlled by a computer with Metrotest 6.0 software, supplied both by Metrotec (Lezo, Spain). Tensile tests were performed at a displacement rate of 6 mm/min. The crosshead displacement and load from the load cell were recorded during the test at a sampling rate of 10 readings per second.

### 2.3. Numerical Models

#### 2.3.1. Finite Element Analysis Model

**Figure 3:**
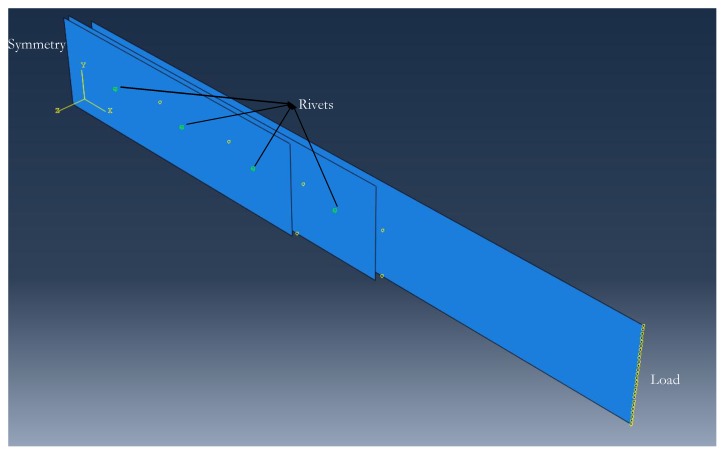
Finite element model in Abaqus CAE of the composite-composite riveted joint with the load and symmetry boundary conditions indicated in their respective edges.

Three-dimensional finite element models of the joints were designed and analyzed in commercial code Abaqus CAE. Only half of each of the lap joints were analyzed due to their symmetry respect to the yz plane (see Figure 3), which reduced computational time significantly. The substrate and doublers were modelled as shells with an offset from the middle plane. Shell elements provide good accuracy and faster solution compared to solid elements, since the former have lower density and require less computational power [28]. Shells are thin 4-node, doubly-curved elements with reduced integration elements (S4R). A refined mesh with quad-dominated medial axis meshing was used for the shells. The contact between surfaces of substrate and doublers was modelled as general surface-to-surface contact. Frictionless and hard contact were used as contact interface properties between composite-composite and composite-metal surfaces. Mesh seed of 1 mm was considered, with the substrate mesh having 4275 elements, doubler 1 mesh having 2700 elements, and doubler 2 mesh having 2075 elements. The numerical models were loaded with identical boundary conditions as in the experiments. FEA was performed using a standard/general algorithm. Force-displacement curves were obtained. To simplify the data extraction from all the nodes along the load edge, a constraint equation was applied. All the nodes along the loading edge were tied to one node, and the output (load vs. displacement) was obtained at this node. Simple models without any joining definitions were used for all the joints. In particular, the joining for riveted, adhesive bonded, and hybrid joints was modelled as follows.

(1) Riveted Joint Model:

The rivets were modelled as point-based fasteners (see Figure 3) in three Cartesian degrees of freedom (x, y, and z axes). Point-based fasteners are mesh independent elements since they are merely points in the model. Use of point-based fasteners is common in the industry due to the large amounts of rivets in aircraft structures and the fact that this fastener model provides sufficiently good accuracy while requiring relatively low computational time [28]. Point-based fasteners in Abaqus are defined by spot weld connections with bushing elements and with stiffness in x, y, and z ($K_{1}$, $K_{2}$, and $K_{3}$ in Equations (1)–(3)) [28]. The fastener radius of influence was 2.5 mm (i.e., the rivet radius in the experiments) with continuum distribution from the doubler top surface to the substrate bottom surface.

(1) $$K_{1}={\left\{\frac{k}{m}\left[{\left(\frac{t_{shell1}+t_{shell2}}{2d}\right)}^{\lambda }\left(\frac{1}{E_{11,shell1}t_{shell1}}+\frac{1}{m\cdot E_{11,shell2}t_{shell2}}+\frac{1}{2\cdot E_{fast}t_{shell1}}+\frac{1}{2m\cdot E_{fast}t_{shell2}}\right)\right]\right\}}^{-1}$$

(2) $$K_{2}={\left\{\frac{k}{m}\left[{\left(\frac{t_{shell1}+t_{shell2}}{2d}\right)}^{\lambda }\left(\frac{1}{E_{22,shell1}t_{shell1}}+\frac{1}{m\cdot E_{22,shell2}t_{shell2}}+\frac{1}{2\cdot E_{fast}t_{shell1}}+\frac{1}{2m\cdot E_{fast}t_{shell2}}\right)\right]\right\}}^{-1}$$

(3) $$K_{3}=\frac{\pi d^{2}E_{fast}}{4\left(t_{shell1}+t_{shell2}\right)}$$

In the above equations, k is 2.2 and λ is 0.4 for solid rivets, m is 1 for single shear lap joints, $t_{shell1}$ and $t_{shell2}$ are the thicknesses of shell 1 and shell 2, and d is the rivet hole diameter. The thickness of shell 1 (at the first rivet row) is the thickness of the substrate plus doubler 1 (4.5 mm for composite-composite and 4.5875 mm for composite-metal joints), whereas, for shell 2 (at the second, third, and fourth rivet rows), it is the sum of the thicknesses of the substrate, doubler 1, and doubler 2 (6 mm for composite-composite and 6.175 mm for composite-metal joints). $E_{11}$, $E_{22},$ and $E_{fast}$ are the elastic moduli of the materials: 72 GPa for the aluminum alloy doublers, 68 GPa for the composite lamina, and 70 GPa for the point-based aluminum fasteners. $G_{fast}$ is the shear modulus of the fastener (27 GPa), and L is the length of the fastener at the corresponding rivet row.

(2) Adhesive Bonded and Hybrid Joint Models:

CZM was used to model the adhesive bonded joints, as in Reference [22]. The adhesive bond between the surfaces of substrate and doublers was generated by a contact interaction. The adhesive was set in Abaqus as elastic perfect plastic. The contact interface has no physical thickness but has the same effect of an adhesive layer of thickness of 0.25 mm. The cohesive contact algorithm was generated between the interfaces of the adhesive surfaces. This algorithm uses traction-separation criteria, giving good accuracy and low error when validated against experimental results [28]. Nominal stresses and nominal strains define the elastic behavior of the cohesive interface across the surfaces, and are represented by three traction components, $t_{n}$, $t_{s}$, and $t_{t}$ (in the normal, shear, and traction directions, respectively), and three nominal separation components, $\delta_{n}$, $\delta_{s}$, and $\delta_{t}$. The nominal strains are defined as the nominal separations divided by the original thickness of the cohesive element $T_{0}$ [28].

(4) $$\varepsilon_{n}=\frac{\delta_{n}}{T_{0}},\;\varepsilon_{s}=\frac{\delta_{s}}{T_{0}},\;\varepsilon_{t}=\frac{\delta_{t}}{T_{0}}$$

The elastic matrix of the cohesive elements is then expressed as the formula below [28].

(5) $$t=\left\{\begin{array}{c} t_{n}\\ t_{s}\\ t_{t} \end{array}\right\}=\left[\begin{array}{ccc} k_{nn} & k_{ns} & k_{nt}\\ k_{sn} & k_{ss} & k_{st}\\ k_{tn} & k_{ts} & k_{tt} \end{array}\right]\left\{\begin{array}{c} \varepsilon_{n}\\ \varepsilon_{s}\\ \varepsilon_{t} \end{array}\right\}=K\varepsilon$$

For uncoupled behavior of traction and separation, the off-diagonal terms are null in the elastic matrix K. The elastic properties of the adhesive were calculated and introduced in terms of $k_{nn}$, $k_{ss}$, and $k_{tt}$, where $k_{nn}$ is the Young’s modulus of the adhesive divided by $T_{0}$, and $k_{ss}$ and $k_{tt}$ are the shear moduli of the adhesive divided by $T_{0}$. In this study, $E_{adhesive}$ and $G_{adhesive}$ is 1 GPa, and the adhesive thickness is 0.25 mm. The elastic perfectly plastic response of the adhesive was set in the numerical analysis, and no damage model was used. Lastly, the hybrid joints were simply modelled by using both the point-based fasteners and the CZM.

#### 2.3.2. Helius Composite Tool Model

Composites are, in general, heterogeneous and anisotropic (their properties in the longitudinal and transversal directions depend on fiber layout and orientation, etc.). The composite substrate used in this work is unique. Thus, a simple model of the CFRE substrate was created in Autodesk Helius composite tool to analyze the ply load distributions, first-ply failure with progressive failure analysis (PFA), and failure envelopes in longitudinal, transversal, and through-thickness directions [30]. When the load is applied to a composite laminate, knowing the load distribution among plies is important for understanding the local stress concentrations for inter-laminar shear [30]. Once the ply load distribution is known, then analysis of first-ply failure can be performed to calculate the maximum stress in the longitudinal, transversal, and shear directions. The initial failure of the ply propagates to other plies. PFA provides ply failure and predicts the mode of failure as either fiber or matrix failure [30]. In this analysis, the substrate consisted of 12 plies with the ply orientation discussed in Section 2.1 and the lamina properties shown in Table 1.

## 3. Results and Discussion

### 3.1. Experimental Results

**Figure 4:**
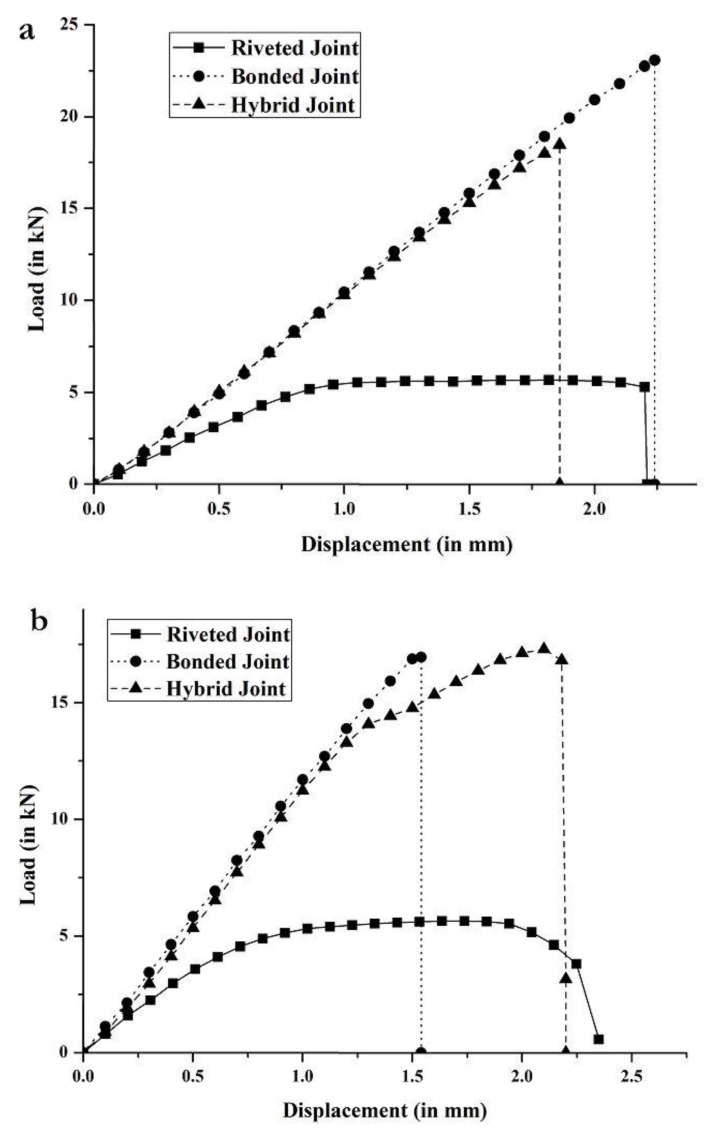
Experimental load vs. displacement curves for riveted, adhesive bonded, and hybrid joints for (**a**) composite-composite and (**b**) composite-metal configurations.

Figure 4 shows the load versus displacement curves obtained from the experiments for composite-composite and composite-metal configurations of riveted, adhesive bonded, and hybrid joints. In this comparative analysis, three specimens of each type of joint are experimentally tested. The peak load of each specimen, as well as the average peak load and standard deviation for all three specimens, as obtained from the experiments for each of the six joint types, are reported in Table 5 and Table 6.

**Figure 5:**
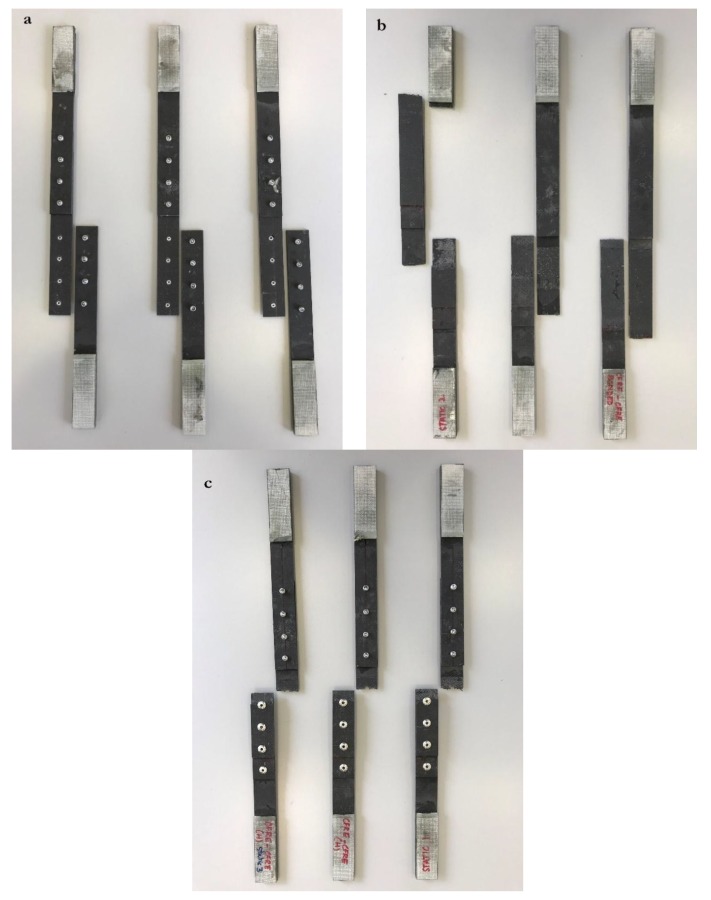
Failure of composite-composite (**a**) riveted, (**b**) adhesive bonded, and (**c**) hybrid joints.

As shown in Figure 4a and Table 5 for composite-composite configuration, riveted joints have the lowest average peak load (5.74 kN), with very low standard deviation (0.02 kN), and peak displacement of 2.1 mm at failure. The failure mode observed for all riveted joints was abrupt failure of the rivets due to rivet shear, which shows that the rivets are the weakest points of these joints and their failure causes the joints to fail (see Figure 5a). The load vs. displacement curve shows elastic and plastic behavior. Linear elasticity is observed up to a load of 4.3 kN, beyond which rivets have sheared plastically up to failure. The rivet load (peak load of the joint divided by the number of rivets) for composite-composite riveted joints is 717 N. The rivet shear strength (rivet load divided by area of the rivet) is 40 MPa. In these preliminary estimations, it is assumed that each rivet transmits the same load. In reality, each rivet does not transmit the same load, but these values provide an estimate of the average load carried by each individual rivet in the joint. Further information about the stress distribution within the riveted joints is shown in Section 3.2.1, as obtained from FEA.

For composite-composite configuration, adhesive bonded joints have the highest average peak load (24.8 kN), with a standard deviation of 1.6 kN (6.5% of the average load, although the specimens were bonded under the same conditions), and peak displacement of 3 mm at failure. Improper or unequal surface treatment or voids in the adhesive bond may have affected specimens, which explains such dispersion in the results. Unlike riveted joints, adhesive bonded joints show only linear elastic behavior. Among the three tested specimens of adhesive bonded joint, two experienced cohesive failures, with traces of adhesive on both substrate and doubler 1 (Figure 5b), while the third specimen showed doubler failure (see center specimen in Figure 5b). The estimated shear strength of the adhesive (peak load of the joint divided by the area of the adhesive bond) is 4.8 MPa. This value is much lower than the fracture strength of the adhesive provided by the manufacturer (21.38 MPa, as shown in Table 4). The reason is that many factors affect the final strength of the adhesive bond, such as the substrate and doubler material, surface finishing, and thickness of the bond.

Lastly, composite-composite hybrid joints have an average peak load of 18.5 kN, with a standard deviation of 0.4 kN (2.2% of the average load). This low dispersion in the results for riveted and hybrid joints compared to adhesive bonded joints may be due to the presence of the rivets, while, for adhesive bonded joints, there is higher dispersion in the results because of uneven performance of the bonds due to differences in adhesive application conditions, defects, and/or voids. Net-section failure is observed in all three specimens (see Figure 5c), which suggests high vulnerability of the composite plates to fastener holes. Recall that, for the riveted joints, net-section failure was not the failure mode but rivet shear. This shows that the strength of the rivets is lower than the strength of the substrate or doubler plates (even if they are composite lamina with drilled holes). However, in the hybrid joints, the strength of the composite plates with the drilled holes is lower than the strength of the adhesive and rivets together. Summarizing, the average peak load of the composite-composite adhesive bonded joints is four and a half times that of riveted joints, and 34% higher than that of hybrid joints.

**Figure 6:**
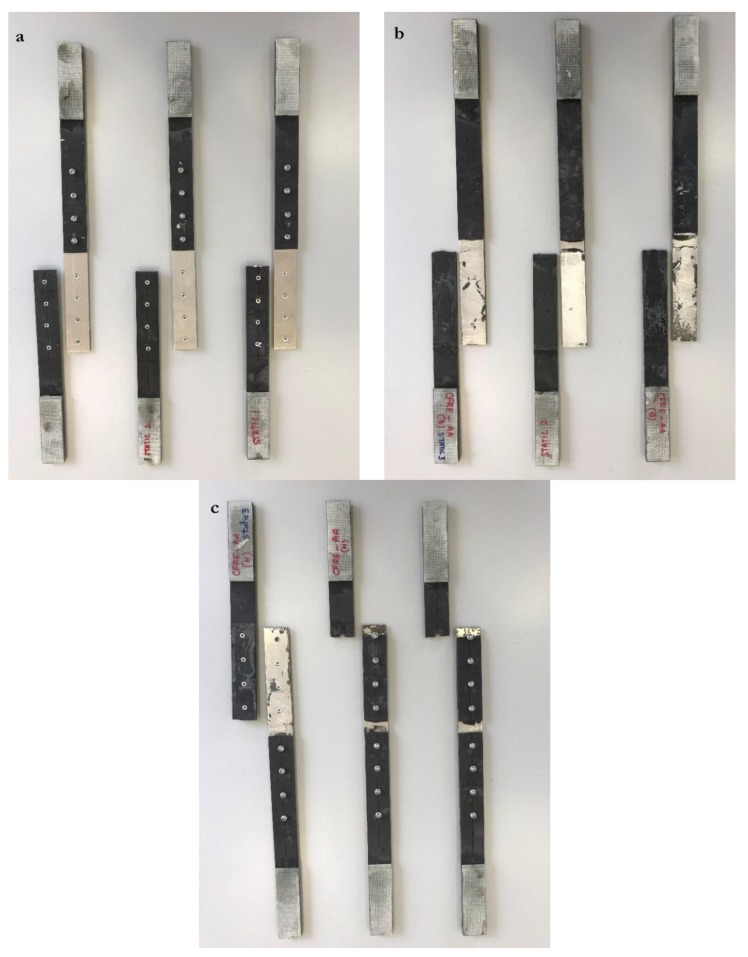
Failure of composite-metal (**a**) riveted, (**b**) adhesive bonded, and (**c**) hybrid joints.

As shown in Figure 4b and Table 6 for composite-metal configuration, riveted joints have an average peak load of 5.6 kN, with a low standard deviation (0.07 kN). Linear elasticity is observed up to displacement of 0.7 mm, which is significantly lower than the elastic limit of the composite-composite riveted joint (see Figure 4a). This may be due to the dissimilar substrate and doubler material in the former, which causes lower stiffness of rivets by effecting the load-transfer between rivet rows. Rivet shear caused the failure of all three riveted joint specimens (see Figure 6a). The rivet load for composite-metal riveted joints is 707 N, and the rivet shear strength is 40 MPa.

For composite-metal adhesive bonded joints, they have an average peak load of 16.9 kN (32% lower than for composite-composite adhesive bonded joints), with a standard deviation of 1 kN (5.9% of the average load). Adhesive failure was the failure mode of all three adhesive bonded joint specimens (see Figure 6b), with the failed adhesive remaining on the substrate. The shear strength of the adhesive is now 3.25 MPa, which is 68% of the shear strength of the adhesive as estimated for the composite-composite adhesive joint. The reduction in shear strength can be due to the adhesive interaction between dissimilar materials [12].

Lastly, composite-metal hybrid joints, with an average peak load of 17.3 kN and standard deviation of 0.6 kN, have slightly higher load capacity than composite-composite adhesive bonded joints (see Figure 6c). Two out of the three composite-metal hybrid joint specimens failed due to net-section failure of the substrate, while the third specimen failed due to cohesive failure and rivet shear. The net-section failure mode suggests vulnerability of composite substrates to high stress concentrations around the rivet holes. The low standard deviation in the strength of the specimens suggests high reliability of hybrid joints for practical applications. When comparing riveted, adhesive bonded, and hybrid joints, riveted joints show the lowest standard deviation, which is followed by hybrid joints. When it comes to applications in the air transport industry, and aviation, in general, reliability of joints is a major concern, but adhesive bonded joints can still be considered. For these, the standard deviation is 5.9% of the average load, and they show high performance. However, proper testing methods for crack detection or debonding play an important role in maintaining standards for these joints.

Energy absorption (EA) is the necessary energy required to break the joints, which is calculated based on the area enclosed by the load vs. displacement curves. The higher the EA, the better the joint static response. Table 7 presents a comparison of the EA for the studied joints, of which composite-composite adhesive bonded joints exhibit the highest value. A simple superposition rule cannot be applied to calculate EA of hybrid joints based on EA of riveted and adhesive bonded joints [31].

From the experimental results, riveted joints show the lowest strength and their strength solely depends on the strength of the rivets. Adhesive bonded joints are better than the riveted joints in a sense that the load is transferred through a wide spread area, which makes their ultimate strength superior to riveted joints, and minimizes the stress concentrations. However, it is worth recalling that the force per unit area transferred by the rivets is higher, which causes high stress concentrations around the rivet holes.

Among adhesive bonded joints, composite-composite joints have strengths that are 47% higher than those of composite-metal joints. This may be due to the similar material used for the substrate and doublers. Lastly, hybrid joints are better than riveted joints. In the case of composite-composite hybrid joints, they have 25% lower strength compared to the adhesive bonded joints. This can be due to the reduction of adhesive bond area (nearly 6%) due to the presence of the rivets. Lastly, among hybrid joints, the strength of the composite-composite joints is 7% higher than that of the composite-metal joints.

### 3.2. Numerical Results

#### 3.2.1. Finite Element Analysis Results

**Figure 7:**
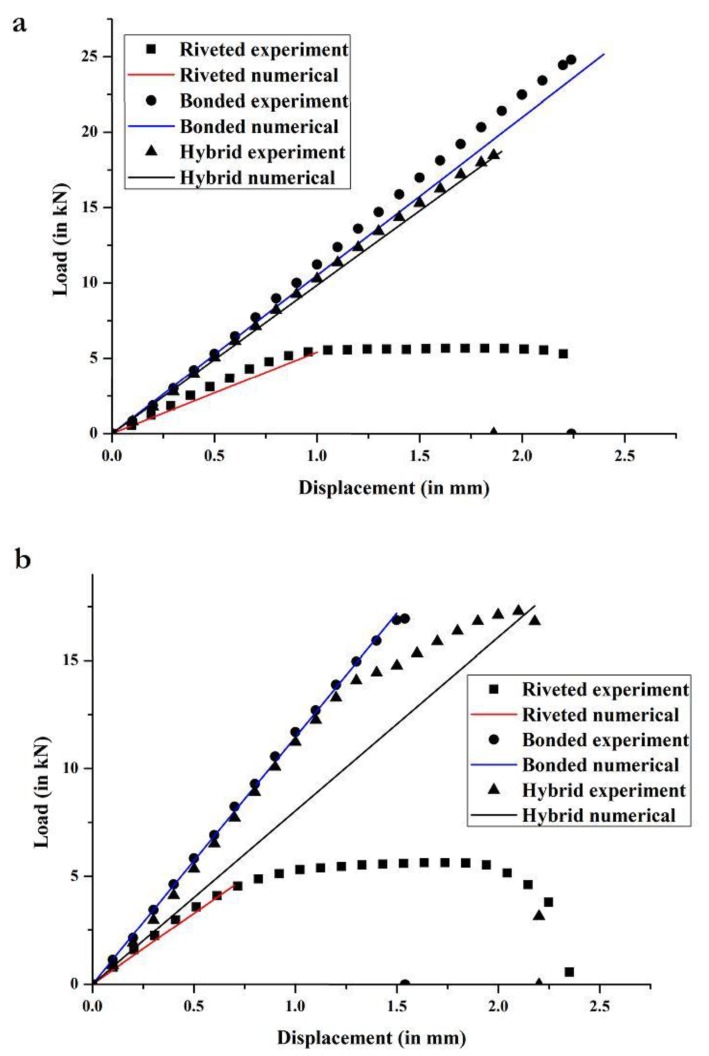
Experimental and numerical load versus displacement curves for riveted, adhesive bonded, and hybrid joints for (**a**) composite-composite and (**b**) composite-metal configurations.

The experimental results are used to validate the results from the commercial FEA code Abaqus. Particularly, the numerical and experimental load versus displacement curves are shown in Figure 7a (for composite-composite joints) and Figure 7b (for composite-metal joints). In the latter figure, note the difference between the experimental and numerical results for hybrid joints. The numerical results tend to be linear, but this is not the case for the experimental results. This may be due to the absence of a plasticity model and failure criteria (linear elastic model) implemented for the rivets and the adhesive layer in the numerical analysis. On the other hand, the dissimilar substrate and doubler material might have caused the elongation of the adhesive and the rivets in the experiments, which explains the large deviation in Figure 7b between the model and experimental results for the composite-metal hybrid joint.

Table 8 shows the error between the numerical and experimental strength or ultimate load for the riveted, adhesive bonded, and hybrid joints of composite-composite and composite-metal configurations. It is remarkable that, in all cases, the error in the peak load at failure displacement is below 1.5%, and the lowest difference is observed for riveted joints, for which the error is smaller than 0.23%.

**Figure 8:**
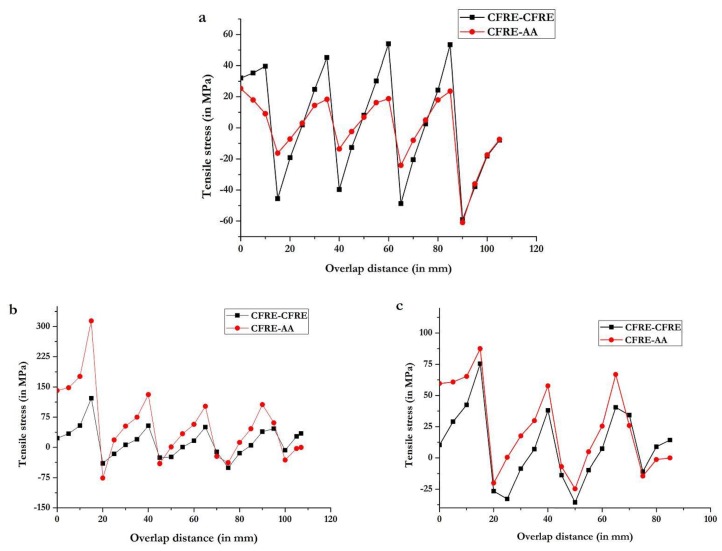
Tensile stress versus the overlap distance for (**a**) substrate, (**b**) doubler 1, and (**c**) doubler 2, for composite-composite and composite-metal riveted joints, at a load of 4.2 kN.

As mentioned earlier, riveted joints are the most commonly used repair technique. This is why the tensile stress response of these joints analyzed in FEA at a load of 4.2 kN is shown in Figure 8, for both composite-composite and composite-metal configurations. The applied load (4.2 kN) is in the elastic stiffness region of the load versus the displacement curve obtained from the experiments. The rivet model explained in Section 2.3.2 is used for obtaining these results, and the overlap distance corresponds to the distance with respect to the yz symmetry plane of the joint.

The tensile stresses in the substrate are significantly lower for the composite-metal riveted joint compared to the composite-composite riveted joint. Each stress peak observed in Figure 8 corresponds to a rivet. Note that each rivet row shows both tensile and compressive stress. The lowest stress is observed at the first rivet row from the symmetry plane, and then the stress increases sequentially up to the fourth rivet row. After the fourth rivet row, the stresses in the substrate for the composite-composite and composite-metal joints are identical.

For doubler 1 (see Figure 8b), high stress values are observed for the composite-metal joint. This may be due to the stress distribution between dissimilar materials, since the CFRE lamina are brittle and have higher stiffness than the metal doublers, which are ductile. For both composite-composite and composite-metal riveted joints, the stresses drop from the first to the fourth rivet row. Particularly, tensile stresses are much higher (nearly three times higher) at the first rivet row compared to the second rivet row.

For doubler 2 (see Figure 8c), the stresses at the first rivet row are higher compared to the second and third rivet rows. Lastly, there is not much difference in the stress in these rivet rows for the composite-composite and composite-metal joints.

From this analysis, it can be seen that the tensile stresses in the substrate with doublers of the same material as the substrate (i.e., CFRE) are higher compared to the stresses in the substrate with doublers of different material (i.e., AA 2024-T3). On the other side, the stresses in the doublers of the same material as the substrate are lower compared to those in the doublers of different material than the substrate. This may be due to the difference in stiffness of substrate and doubler materials, with metals being more ductile and composites being stiffer. There is no large proportion of tensile stress in the substrate at the second, third, and fourth rivet rows with either composite or metal doublers (maximum difference is 40 MPa), but the first rivet row for metal doubler 1 has a nearly three times higher value of tensile stress compared to composite doubler 1. Thus, it can be concluded that the composite substrate with composite doublers shows more homogeneous tensile stress distribution.

#### 3.2.2. Helius Composite Tool Results

**Figure 9:**
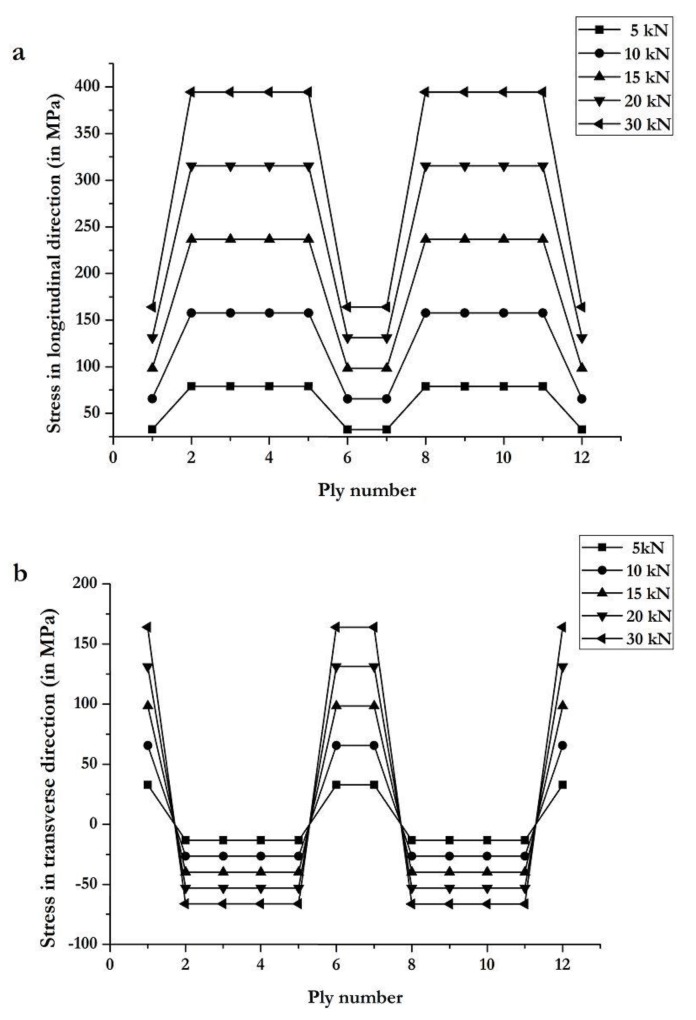
Ply stress distribution computed with Autodesk Helius tool for composite substrate along (**a**) the longitudinal direction and (**b**) the transverse direction.

In the joint designs, not only the joining method but also the substrate material is important, since it is the primary structural element. CFRE substrates are more complicated since the properties depend significantly on the orientation of the plies. Hence, it is important to analyze the CFRE substrate used in this study. For this purpose, the Autodesk Helius composite tool is used to compute the composite substrate stress distribution, ply failure, and failure mode. The stress distributions obtained with Helius in the longitudinal and transverse directions for the substrate plies are shown in Figure 9. It can be noticed that the longitudinal stress is the highest in 0/90 plies (plies 2, 3, 4, 5, 8, 9, 10, and 11) and the transverse stress is the highest in 45/135 plies (plies 1, 6, 7, and 12).

The composite substrate is analyzed for various loads: 5, 10, 15, 20, and 30 kN. From Figure 9, it is observed that plies in positions 2, 3, 4, 5, 8, 9, 10, and 11 have the highest stress in the loading direction. Low stress is observed in outer plies (positions 1 and 12) and middle plies (positions 6 and 7), which have orientations 45/135, are not aligned with the loading direction. Figure 9b shows the ply stress distribution in the transverse direction. It is noticed that 45/135 plies have positive stress, while 0/90 plies have negative stress, which indicates compression of these plies.

**Figure 10:**
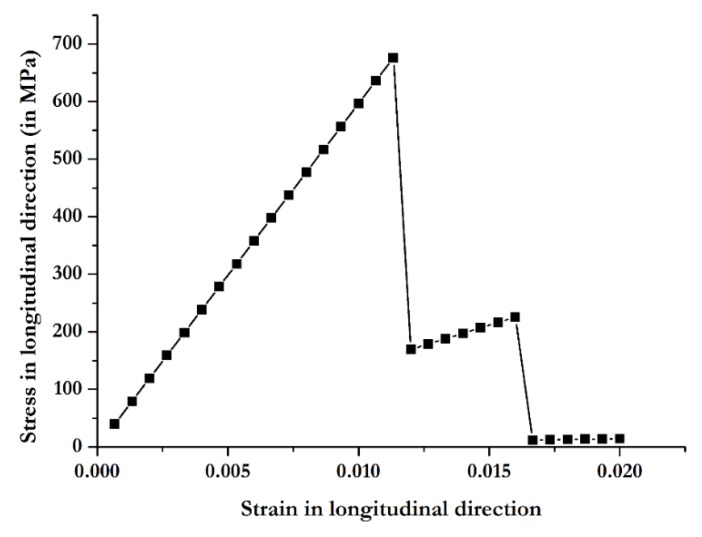
Longitudinal stress versus strain, as computed with Autodesk Helius tool for the composite substrate.

The stress distribution in the plies provides information about ply load distribution but not about their failure mode. Hence, PFA is done with Helius to understand the failure mode of the substrate plies. Figure 10 shows the PFA results, which indicates that the first failure occurs in plies 2, 3, 4, 5, 8, 9, 10, and 11 (plies with 0/90 orientation) at 678 MPa at a strain of 0.011. Then, the stress drops to 156 MPa. From 156 MPa, plies 1, 6, 7, and 12 (plies with 45/135 orientation) take stress up to 226 MPa, and then the substrate fails completely. Failure of 0/90 plies is caused by fiber failure, while, for 45/135 plies, it is due to matrix failure. Christensen’s failure criterion is used in PFA. According to it, either fiber failure or matrix failure can occur, as determined by Equations (6) and (7), respectively [32].

(6) $$\left(\frac{1}{S_{22}^{+}}-\frac{1}{S_{22}^{-}}\right)\left(\sigma_{22}+\sigma_{33}\right)+\frac{1}{S_{22}^{+}S_{22}^{-}}{\left(\sigma_{22}+\sigma_{33}\right)}^{2}+\frac{1}{S_{23}^{2}}\left(\sigma_{23}^{2}-\sigma_{22}\sigma_{33}\right)+\frac{1}{S_{12}^{2}}\left(\sigma_{12}^{2}+\sigma_{13}^{2}\right)\geq 1.0$$

where $S_{23}\geq \frac{1}{2}\sqrt{S_{22}^{+}+S_{22}^{-}}$ and:

(7) $$\left(\frac{1}{S_{11}^{+}}-\frac{1}{S_{11}^{-}}\right)\sigma_{11}+\frac{\sigma_{11}^{2}}{S_{11}^{+}S_{11}^{-}}\geq 1.0$$

where $S_{11}^{+}$ and $S_{11}^{-}$ are the values of $\sigma_{11}$ in longitudinal tensile and compressive failure, $S_{22}^{+}$ and $S_{22}^{-}$ are the values of $\sigma_{22}$ in transverse tensile and compressive failure, and $S_{12}$ and $S_{23}$ are the absolute values of $\sigma_{12}$ and $\sigma_{23}$ in the shear direction. The mechanical properties of the composite substrate shown in Table 1 are used in Christensen’s failure criterion equations [32].

**Figure 11:**
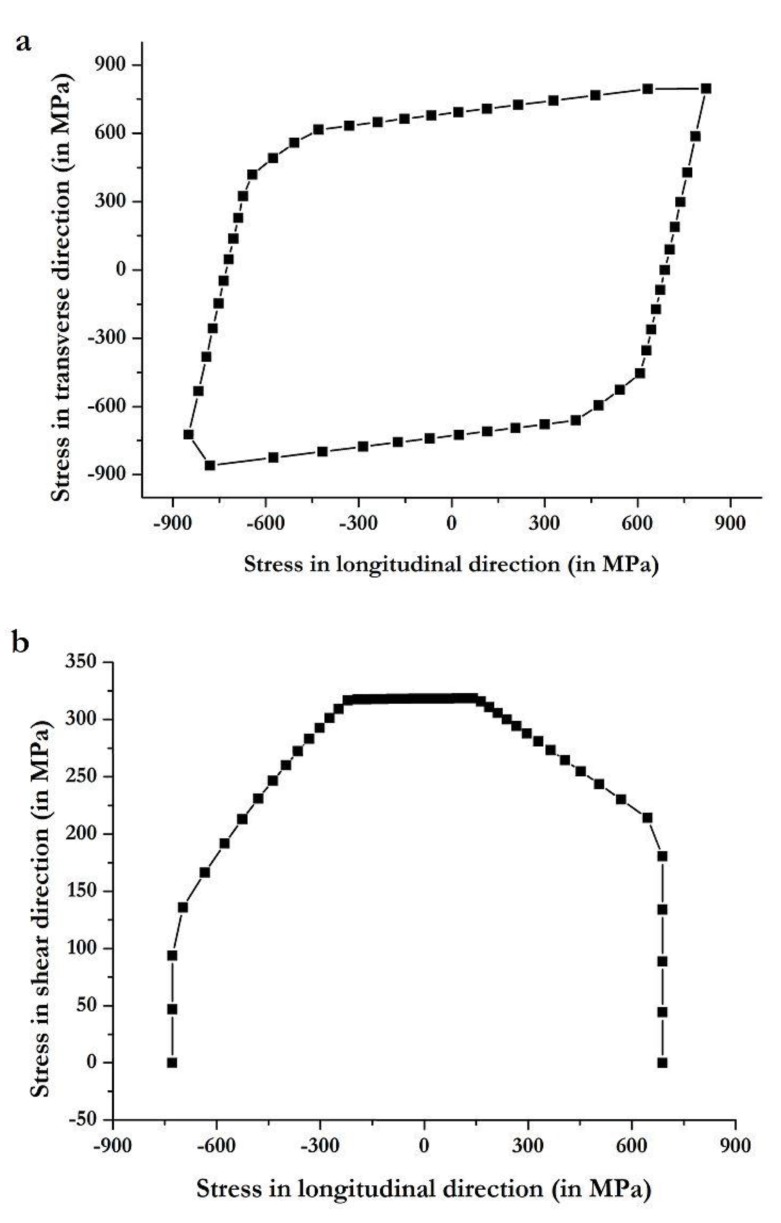
Failure envelope of the composite substrate in (**a**) transverse versus longitudinal direction, and (**b**) shear versus longitudinal direction, as computed with the Autodesk Helius tool.

The failure envelopes for the substrate provide the limits of stress in the longitudinal, transverse, and shear directions. Figure 11a shows the $S_{11}$ versus $S_{22}$ envelope plot for the composite substrate, while Figure 11b shows the $S_{11}$ versus $S_{12}$ envelope plot. These plots provide complete information of the stress in the substrate along the main loading direction compared to other directions.

## 4. Conclusions

This work presents an experimental and numerical analysis of composite substrates repaired with composite and aluminum alloy doublers, with riveted, adhesive bonded, and hybrid joints. The results from this investigation lead to the following conclusions.

- Riveted joints have the lowest strength (although the force per unit area transferred by the rivets is higher than the adhesive bond), but the test results for strength show very low dispersion (negligible standard deviation), and, thus, the results seem to be very reliable and precise.
- Adhesive bonded joints show the highest strength both for composite-composite and composite-metal joints. However, these joints show the highest standard deviation in strength test results, around 6.5% of the average peak load, which is considerable. The likely reasons are differences in adhesive application conditions and the presence of defects.
- Two out of three specimens of composite-composite adhesive bonded joints showed cohesive failure mode with traces of adhesive on both substrate and doubler 1, while the third specimen failed due to doubler failure.
- All the specimens of composite-metal adhesive bonded joints failed due to adhesive failure. The failed adhesive remained attached to the composite substrate rather than the metal doubler.
- The strength of hybrid joints is relatively better than that of riveted joints, and the strength results for hybrid joints show lower standard deviation compared to adhesive bonded joints. It seems that the rivets mitigate the dispersion caused by the reasons exposed above.
- For hybrid joints, three specimens of composite-composite and two specimens of composite-metal failed due to net-section failure. The third specimen of composite-metal hybrid joint failed due to adhesive and rivet shear failure. For composite-composite hybrid joints, the composite doublers failed, while, for composite-metal hybrid joints, the composite substrate failed. This suggests vulnerability of composite structures to mechanical fastener holes, which is one of the most important findings of this work. For the riveted joints, net-section failure was not the failure mode but rivet shear. This shows that the strength of the rivets is lower than the strength of the substrate or doubler plates (even if they are composite lamina with drilled holes). However, in the hybrid joints, the strength of the composite plates with the drilled holes is lower than the strength of the adhesive and rivets together.
- The experimental results are used to validate the results from the commercial FEA code Abaqus. It is remarkable that, in all cases, the difference between the experimental and numerical peak load at failure displacement is below 1.5%, and the lowest difference is observed for riveted joints, for which the error is smaller than 0.23%.
- Progressive Failure Analysis of carbon composite substrate using the Autodesk Helius composite tool suggests that the initial failure of substrate occurs at 678 MPa in 0/90 plies and that final failure of the substrate occurs at 226 MPa in 45/135 plies.

## Figures and Tables

**Table 1:** Mechanical properties of the carbon fiber reinforce epoxy (CFRE) lamina, as provided by the manufacturer (Composites Ate, S.L., Barcelona, Spain).

Composite	*E*_11_ (GPa)	*E*_22_ (GPa)	*G*_12_ (GPa)	*G*_13_ (GPa)	*ν*_12_ (-)	Tensile Strength (MPa)			Compressive Strength (MPa)	
						*+S*_11_	*+S*_22_	*+S*_12_	*−S*_11_	*−S*_22_
CFRE	67.6	67.6	4.2	4.2	0.04	885	885	97	835	835

**Table 2:** Mechanical properties of the aluminum alloy 2024-T3, as provided by the manufacturer (Kaiser Aluminum, Spokane, WA, USA).

Al Alloy	Yield Stress	UTS	% Area Reduction	Brinell Hardness
2024-T3	316 MPa	464 MPa	20.2%	HB 123

**Table 3:** Chemical composition of the aluminum alloy 2024-T3, as provided by the manufacturer (Kaiser Aluminum, Spokane, WA, USA).

Al Alloy	Units	Si	Fe	Cu	Mn	Mg	Zn	Ti	Cr	Al
2024-T3	wt.%	0.50	0.50	4.90	0.90	1.80	0.25	0.15	0.10	90.90
2024-T3	at.%	0.50	0.25	2.16	0.46	2. 70	0.11	0.09	0. 05	94.31

**Table 4:** Mechanical properties of the adhesive Araldite 2031, as provided by the manufacturer (Huntsman Advanced Materials GmbH, Basel, Switzerland).

Adhesive	*E* (GPa)	$\sigma_{fracture}\;(\mathrm{MPa})$	$\varepsilon_{fracture}\;(\%)$
Araldite 2031	1.057	21.38	6.39

**Table 5:** Experimental results for strength of riveted, adhesive bonded, and hybrid joints with composite substrate and composite doublers.

Joint Type	Specimen 1 (kN)	Specimen 2 (kN)	Specimen 3 (kN)	Average (kN)	Standard Deviation	
					(kN)	(%)
Riveted joint	5.72	5.75	5.73	5.74	0.02	0.4
Bonded joint	26.0	25.4	23.0	24.8	1.6	6.5
Hybrid joint	18.1	19.0	18.3	18.5	0.4	2.2

**Table 6:** Experimental results for strength of riveted, adhesive bonded, and hybrid joints with composite substrate and metal doublers.

Joint Type	Specimen 1 (kN)	Specimen 2 (kN)	Specimen 3 (kN)	Average (kN)	Standard Deviation	
					(kN)	(%)
Riveted joint	5.53	5.65	5.65	5.61	0.07	1.3
Bonded joint	17.3	17.7	15.8	16.9	1.02	5.9
Hybrid joint	17.6	16.6	17.7	17.3	0.6	3.5

**Table 7:** Experimental results for energy absorption (EA) by the riveted, adhesive bonded, and hybrid joints in composite-composite and composite-metal configurations.

Joint Type	Composite-Composite EA (J)	Composite-Metal EA (J)
Riveted joint	9.9	10.1
Bonded joint	28	13.7
Hybrid joint	17.6	23.4

**Table 8:** Strength of the studied joints as obtained from numerical analysis and experiments, and error between numerical and experimental results.

Joint Type		Numerical Strength (kN)	Experimental Strength (kN)	% Difference
CFRE-CFRE	Riveted joint	5.2	5.10	0.2
	Bonded joint	25.2	24.8	1.3
	Hybrid joint	18.7	18.5	1.4
CFRE-2024-T3	Riveted joint	4.9	4.6	0.9
	Bonded joint	17.2	17.0	1.5
	Hybrid joint	17.5	17.3	1.4
